# Epidemiology, serovar distribution and spatiotemporal patterns of Salmonella foodborne infections in Liaoning Province, China, 2014–2024: an analysis of sentinel surveillance data

**DOI:** 10.3389/fpubh.2026.1708582

**Published:** 2026-02-23

**Authors:** Xinling Yu, Xiangyun Liu, Xiaoxiao Du, Yiming Pei, Kailin Wang, Hao Zhang, Wenli Diao

**Affiliations:** 1 Institute of Preventive Medicine, China Medical University, Shenyang, Liaoning, China; 2 China Medical University, Shenyang, Liaoning, China; 3 Liaoning Provincial Center for Disease Control and Prevention, Shenyang, Liaoning, China

**Keywords:** epidemiology, foodborne disease, Salmonella, spatial autocorrelation, spatiotemporal analysis

## Abstract

**Objective:**

Using long-term data from hospital-based sentinel (active) surveillance of foodborne diseases in Liaoning Province from 2014 to 2024, we examined temporal trends, seasonality, the serovar spectrum, and city-level spatiotemporal distribution of salmonellosis to inform optimization of surveillance and targeted prevention and control. This study provides one of the longest provincial-level sentinel-based spatiotemporal analyses of salmonellosis in China.

**Methods:**

Laboratory-confirmed infections with *Salmonella enterica* reported by sentinel hospitals in Liaoning Province were extracted from the National Foodborne Disease Surveillance System. The Mann–Kendall test was used to evaluate monotonic trends in annual positivity rates. Monthly seasonal indices were calculated to describe seasonality. At the city level, a first-order queen contiguity spatial weight matrix was constructed to perform global and local Moran’s *I* analyses, with LISA results adjusted using the Benjamini–Hochberg false discovery rate (FDR) procedure. Spatiotemporal clusters were identified using SaTScan with a discrete Poisson model.

**Results:**

From 2014 to 2024, 38,810 cases were tested and 346 were positive for *S. enterica*, yielding an overall positivity rate of 0.89%. The positivity rate showed a significant, gradual upward trend (*z* = 2.49, *p* < 0.05). A unimodal seasonal pattern was observed, with high-risk months from June to August and a peak in July. Young children (particularly one-year-olds and children cared for at home) accounted for the largest proportion of detected cases. The predominant serovars were *S. enterica* serovar Enteritidis and *S. enterica* serovar Typhimurium. Global spatial autocorrelation was not significant; however, local analyses indicated that Chaoyang City remained a stable hotspot after FDR correction. SaTScan identified a Class I (most likely) spatiotemporal cluster centered on Chaoyang City for each predominant serovar (both *p* < 0.001).

**Conclusion:**

Sentinel surveillance in Liaoning Province suggests a year-by-year increase in the detection level of Salmonella, with summer–early autumn and young children representing key risk windows. Chaoyang City consistently showed a relatively high burden in both local spatial and spatiotemporal analyses. These findings provide evidence for targeted surveillance optimization and risk-based intervention strategies in high-risk seasons, populations, and geographic areas.

## Introduction

Foodborne salmonellosis is widespread globally. It has been estimated that approximately 93.757 million diarrheal episodes and 155,000 deaths occur annually due to salmonellosis worldwide (1). In the United States, salmonellosis remains a major public health concern, with an estimated 0.8–4.0 million infections each year (2), and it is among the leading causes of hospitalization due to foodborne diseases (3). In England and Wales, approximately 73,193 salmonellosis cases are reported annually, ranking third among major bacterial enteric pathogens and contributing substantially to the case-fatality burden (4). According to current taxonomy, most human salmonellosis is caused by *Salmonella enterica*, and clinically important serovars include Enteritidis and Typhimurium, which are defined based on antigenic structures. Despite the increasing availability of foodborne disease surveillance data in China, long-term provincial-level analyses that integrate serovar distribution with spatial and spatiotemporal methods remain limited. Therefore, this study aimed to characterize the epidemiological features and serovar spectrum of foodborne salmonellosis detected through sentinel surveillance in Liaoning Province during 2014–2024, and to assess its temporal trends, seasonality, and city-level spatial and spatiotemporal patterns.

## Materials and methods

### Data source

Data were obtained from the National Foodborne Disease Surveillance System, including surveillance records reported between 2014 and 2024 from actively monitored hospitals (sentinel hospitals) in Liaoning Province. According to the national surveillance protocol, Liaoning selected a number of general hospitals, specialized hospitals and children’s hospitals as sentinel sites, covering different regions and levels of medical care, and conducted surveillance using unified case definitions and laboratory standards.

“Actively monitored hospitals” refer to medical institutions included in the provincial food safety risk surveillance program that continuously collect cases and conduct laboratory testing according to the national protocol. Hospitals must meet all of the following criteria before inclusion:

1. Possess a standardized reporting system for diarrheal diseases and be able to register cases and collect epidemiological information as required by the surveillance protocol;
2. Have laboratory capacity for independent testing of stool or rectal swab specimens, including isolation and culture of Salmonella and serotyping (or the ability to send specimens to designated laboratories for testing);
3. Provide continuous and complete surveillance data and upload case and laboratory records in a timely manner according to the annual surveillance plan;
4. Cover the major catchment population in the area and meet a minimum scale or representativeness requirement to ensure comparability and stability of surveillance data.

In this study, the terms “actively monitored hospitals” and “sentinel hospitals” are used interchangeably to refer to medical institutions participating in the national foodborne disease sentinel surveillance system.

Each year, provincial authorities review candidate hospitals proposed by each city under the National Food Safety Risk Surveillance Plan and finalize the list of sentinel hospitals. All data used in this study were derived from actively monitored hospitals that met the above criteria. We collected and analyzed foodborne disease cases reported by these hospitals from 2014 to 2024, and identified those confirmed by laboratory testing as Salmonella infections. Surveillance case definition: outpatients presenting to intestinal clinics with diarrhea as the main complaint, suspected or reported to be associated with food consumption, passing stool three times or more per day with abnormal stool characteristics (loose, watery, bloody stool). For cases meeting the definition, stool or blood specimens were collected for laboratory testing for Salmonella. Because the surveillance system is based on sentinel sites, the number of cases and the proportion of tested patients may vary by year and by hospital. The “positivity rate” reported in this study therefore reflects only the positivity rate among tested cases in the sentinel surveillance system and cannot be directly interpreted as the incidence in the general population of Liaoning Province.

The data were derived from outpatients attending sentinel hospitals and undergoing specimen testing, representing surveillance among healthcare-seeking individuals rather than population-based incidence monitoring. Sentinel findings may be influenced by the distribution of healthcare resources, access to care, clinical indications for testing, specimen collection practices, and laboratory capacity. In addition, sentinel site configuration may change across years, potentially leading to year-to-year variation in testing volume and positivity. Accordingly, in interpreting results we treat positivity rates and case counts as signals of detection within the sentinel system for describing temporal trends and spatiotemporal heterogeneity, rather than as direct estimates of true incidence. Future work will attempt to incorporate additional information on sentinel site composition and coverage to improve interpretability and generalizability.

### Laboratory testing and serotyping

#### Case information collection

Clinicians in sentinel hospitals collected demographic information (sex, age, occupation, place of residence), date of consultation, clinical symptoms, department of consultation, and suspected food exposures from patients meeting the surveillance case definition. Data were reported through the network system and underwent multi-level review. Centers for Disease Control and Prevention (CDC) at different levels performed data quality control, including checking for missing or logically inconsistent key variables and verifying or excluding problematic records.

#### Laboratory testing

Laboratory testing and serotyping for Salmonella were conducted in sentinel hospitals according to the methods specified in the annual Foodborne Disease Surveillance Manual issued by the China National Center for Food Safety Risk Assessment. In some hospitals and in early years of surveillance, only “Salmonella-positive” or serogroup information was reported, without further identification to specific serovars.

### Statistical analysis

Because sentinel surveillance lacks stable and granular population denominators at the hospital level, population-based incidence rates could not be reliably estimated. Therefore, case counts and positivity rates were used as proxies for detection intensity within the sentinel system. For SaTScan, we used annual time units because the overall number of positive cases was limited; monthly or quarterly aggregation could introduce substantial random fluctuation and reduce the stability of cluster detection.

Annual aggregation smooths short-term variation and is more suitable for identifying long-term patterns and relatively stable spatiotemporal signals. Spatial autocorrelation analyses used a first-order queen contiguity weight matrix to reflect the assumption that geographically adjacent areas may share spatially correlated factors, while acknowledging that real-world transmission networks may not strictly follow administrative adjacency.

Local Moran’s *I* (LISA) involves multiple hypothesis tests across spatial units, which increases the risk of false positives. Therefore, the Benjamini–Hochberg procedure was applied to control the false discovery rate (FDR = 0.05). SaTScan cluster significance was assessed using Monte Carlo randomization within the scanning framework; thus, no additional FDR correction was applied to SaTScan results.

#### Trend analysis

Data were cleaned and analyzed using R software. Annual Salmonella positivity rate among tested foodborne disease cases was calculated and described as a time series. The Mann–Kendall non-parametric test was used to assess the presence of a monotonic temporal trend in positivity rate between 2014 and 2024. The significance level was set at *α* = 0.05.

#### Seasonal analysis

Monthly case counts of Salmonella-positive cases were aggregated for 2014–2024. Seasonal indices were calculated as the ratio of the average number of cases in a given month to the overall monthly average across the year. Based on the seasonal index, four seasonal risk levels were defined: seasonal index ≥ 1.50 as “very high risk period,” 1.20–1.49 as “high risk period,” 0.80–1.19 as “moderate risk period,” and <0.80 as “low risk period.”

#### Spatial autocorrelation analysis

The 14 prefecture-level cities were used as spatial units. GeoDa was used to construct a first-order queen contiguity spatial weight matrix *W* with row standardization.

Global Moran’s *I* was used to assess overall spatial dependence. Values range from −1 to 1; values near 0 indicate spatial randomness, positive values indicate positive spatial autocorrelation, and negative values indicate negative spatial autocorrelation. Moran’s *I*, *Z*-scores, and *p*-values were obtained using GeoDa. Statistical significance was defined as |*Z*| > 1.96 and *p* < 0.05 (5). LISA was used to identify local spatial clusters, categorized as high–high, low–low, high–low, and low–high patterns (6). Map base layers were obtained from the Standard Map Service of the Ministry of Natural Resources of China. Because global indicators capture overall spatial dependence whereas local indicators identify spatial heterogeneity and localized high/low-risk areas, local clustering may still exist even when global Moran’s *I* is not significant, particularly for diseases with heterogeneous or multi-centered distributions. Accordingly, local clusters were interpreted as localized hotspots or anomalous areas rather than province-wide clustering. To control for multiple testing across cities, Benjamini–Hochberg FDR correction was applied to the raw LISA *p*-values (FDR threshold = 0.05). GeoDa permutation testing (999 permutations) was used to obtain empirical distributions and compute *p*-values (*α* = 0.05).

#### Spatiotemporal scan analysis

SaTScan v10.1.2 was used to conduct spatiotemporal scanning based on a discrete Poisson model. For each year, cases from January to December were aggregated into annual totals to form an annual time series. The annual resolution was selected because the number of cases in sentinel hospitals was limited; monthly or weekly counts would be more sensitive to random fluctuation and could compromise inference. Annual aggregation provides a more stable description of long-term patterns.

Prefecture-level cities were used as spatial units and years as temporal units. City-specific annual population counts served as the baseline for expected cases. We used city-level census population as an approximate baseline required by the Poisson model; however, it does not represent sentinel hospital catchment populations, and results should be interpreted as clustering in detection intensity rather than population incidence. The scanning window was circular, and the maximum spatial window size was set to 30% of the total population. Although SaTScan defaults to 50%, previous studies suggest that large windows may yield overly broad clusters that merge smaller hotspots and reduce the ability to identify localized high-risk areas (7). Simulation studies recommend intermediate values (e.g., 30%) as a balance between hotspot focus and spatial coverage (8). Therefore, we used 30% to reduce excessive cluster expansion while retaining detection sensitivity. The window with the maximum log likelihood ratio (LLR) and *p* < 0.05 was defined as the Class I (most likely) cluster; other significant windows were defined as Class II clusters (9). Observed case counts and relative risk (RR) were recorded for interpretation. *p*-values were calculated using Monte Carlo simulations, and RR, LLR, and observed counts were reported.

## Results

### Positivity rate of Salmonella among foodborne disease cases

From 2014 to 2024, a total of 38,810 foodborne disease cases in sentinel hospitals in Liaoning Province underwent laboratory testing for Salmonella, among whom 346 were positive, yielding an overall positivity rate of 0.89% (Table 1). Annual positivity rate was highest in 2021 (1.89%) and lowest in 2016 (0.27%). Mann–Kendall trend analysis of annual Salmonella positivity rate indicated a statistically significant increasing trend over the study period (*z* = 2.491, *p* < 0.05), suggesting a gradual rise in Salmonella positivity rate within the sentinel surveillance system.

**Table 1:** Numbers and positivity rate of foodborne salmonellosis in sentinel hospitals in Liaoning Province, 2014–2024.

Year	Number of detected cases	Number of Salmonella-positive cases	Positivity rate (%)
2014	1854	7	0.38
2015	3,614	14	0.39
2016	4,087	11	0.27
2017	3,943	22	0.56
2018	3,507	30	0.86
2019	3,186	27	0.85
2020	1932	21	1.09
2021	3,011	57	1.89
2022	3,264	53	1.62
2023	4,864	50	1.03
2024	5,548	54	0.97
Total	38,810	346	0.89

### Demographic characteristics and seasonal distribution

Among the 346 Salmonella-positive cases, 183 were male (52.80%) and 163 were female (47.20%). The largest age group was children aged 1 year (14.74%, 51/346), followed by children aged 2–9 years (11.56%, 40/346) and infants under 1 year (4.34%, 15/346). By occupation, children cared for at home accounted for the highest proportion (29.10%, 101/346), followed by retired persons (16.47%, 57/346). These findings suggest that young children, especially those mainly cared for at home, were the primary susceptible group for foodborne salmonellosis in the sentinel system.

Seasonal index analysis revealed a distinct single-peak seasonal pattern. Very high-risk periods were observed from June to August, with the highest seasonal index in July. May and September were high-risk periods, January-February were low-risk periods, and the remaining months were mostly at moderate risk.

### Suspected food exposure

Among the 346 positive cases, 293 (84.68%) reported a single category of suspected food exposure, 7 (2.02%) reported two or more food categories, 34 (9.83%) reported mixed foods, 9 (2.60%) reported unknown foods, and 3 (0.87%) had missing information (Table 2).

**Table 2:** Reported suspected food exposures among patients with foodborne salmonellosis in Liaoning Province.

Exposed food	Number of cases	Proportion (%)
Single food type	293	84.68
Two or more food types	7	2.02
Mixed foods	34	9.83
Unknown foods	9	2.60
Not reported	3	0.87
Total	346	100

Among the 293 cases reporting a single exposure category, the most frequently reported categories were fruits and fruit products (64, 21.84%), cereals and cereal products (63, 21.50%), meat and meat products (47, 16.04%), and aquatic animal products (32, 10.92%). Together, these accounted for 70.30% (Table 3). These findings suggest that suspected exposures mainly involved commonly consumed foods and may be associated with food handling, storage, and cross-contamination risks. However, because exposure information was self-reported, it should be interpreted as descriptive signals rather than causal evidence. This pattern may reflect reporting bias and the high frequency of consumption of these foods rather than true etiological attribution.

**Table 3:** Single-category suspected food exposures among patients with foodborne salmonellosis in Liaoning Province.

Categories of exposed foods	Number of cases	Proportion (%)
Fruits and their products	64	21.84
Cereals and their products	63	21.50
Meat and meat products	47	16.04
Aquatic animals and their products	32	10.92
Vegetables and their products	28	9.56
Milk and dairy products	19	6.48
Eggs and egg products	11	3.75
Beverages and frozen drinks	10	3.41
Beans and bean products	8	2.73
Infant food	4	1.37
Other foods	4	1.37
Nuts and their products	1	0.34
Oils and fats	1	0.34
Condiments	1	0.34
Total	293	100.00

### Specimen collection

All 346 cases had biological specimens collected. Stool specimens were collected from 331 cases (95.66%), blood specimens from 14 cases (4.05%), and both stool and blood specimens from 1 case (0.29%).

### Serovar distribution

A total of 346 cases were included, of which 127 (36.71%) had definitive single-serovar identification, comprising 13 serovars. The remaining cases were reported only as “Salmonella positive” or with serogroup information and could not be resolved to a single serovar; these cases were excluded from serovar composition analyses. Among the 127 cases with a single serovar identified, *S. enterica* serovar Enteritidis was the most common (53 cases, 41.73%), followed by *S. enterica* serovar Typhimurium (36 cases, 28.35%) (Table 4).

**Table 4:** Distribution of single Salmonella serovars among fully typed isolates from patients with foodborne salmonellosis in Liaoning Province (*n* = 127).

Serovar	Number of cases	Proportion (%)
*S. enterica* serovar Enteritidis	53	41.73
*S. enterica* serovar Typhimurium	36	28.35
*S. enterica* serovar London	9	7.09
*S. enterica* serovar Paratyphi A	6	4.72
*S. enterica* serovar Stanley	5	3.94
*S. enterica* serovar Paratyphi B	5	3.94
*S. enterica* serovar Agona	3	2.36
*S. enterica* serovar Infantis	3	2.36
*S. enterica* serovar Dublin	2	1.57
*S. enterica* serovar Rissen	2	1.57
*S. enterica* serovar Thompson	1	0.79
*S. enterica* serovar Typhi	1	0.79
*S. enterica* serovar Bovismorbificans	1	0.79
Total	127	100

Proportions use *n* = 127 as the denominator; cases without single-serovar identification were excluded.

Across years, these two serovars remained frequently detected. However, because most cases lacked definitive serovar identification and because sentinel site configuration and serotyping capacity may differ across years, temporal variation in serovar distribution is presented descriptively and is not interpreted as evidence of etiological shifts.

### Spatial autocorrelation analysis

#### *Salmonella enterica* serovar Enteritidis

Global spatial autocorrelation analysis showed that the city-level distribution of *S. enterica* serovar Enteritidis case counts during 2014–2024 was not significant (Moran’s *I* = −0.26, *Z* = −1.30, *p* > 0.05), indicating no evidence of overall spatial clustering at the prefecture-city level.

The LISA based on case counts suggested that Shenyang and Dalian had a tendency toward high–high clustering (local Moran’s *I* = 0.29 and 0.26, respectively), but neither remained significant after FDR correction (*q* > 0.05), indicating only weak clustering signals. In contrast, Chaoyang City remained significant after Benjamini–Hochberg adjustment (*q* = 0.048 < 0.05) and was classified as a high–high cluster, suggesting that Chaoyang City was the main spatial hotspot.

#### *Salmonella enterica* serovar Typhimurium

Global spatial autocorrelation for *S. enterica* serovar Typhimurium case counts during 2014–2024 was also not significant (Moran’s *I* = −0.26, *Z* = −1.59, *p* > 0.05). Before multiple-testing correction, several cities (e.g., Chaoyang, Shenyang, Anshan, Dandong, and Huludao) showed high-value clustering patterns in some years, whereas most other cities showed low values or random distributions. After Benjamini–Hochberg FDR correction (FDR = 0.05), only Chaoyang City remained statistically significant (adjusted *p* < 0.05). Overall, these results indicate no widespread significant spatial clustering at the city level across Liaoning Province, but a robust local high-value clustering signal in Chaoyang City.

### Spatiotemporal scan analysis

Using annual cycles, SaTScan identified one spatiotemporal cluster for *S. enterica* serovar Enteritidis: a Class I cluster consisting solely of Chaoyang City, with a radius of 0.00 km and LLR = 47.17 (Table 5). Similarly, one Class I cluster was identified for *S. enterica* serovar Typhimurium: Chaoyang City alone, with a radius of 0.00 km and LLR = 17.35 (Table 6).

**Table 5:** Annual spatiotemporal scan results for *S. enterica* serovar Enteritidis infections in Liaoning Province, 2014–2024.

Cluster type	Cluster city	Occurrence time (year)	Cluster radius (km)	RR value	LLR value	*p*-value
Class I	Chaoyang city	2014–2024	0	21.13	47.17	<0.001

**Table 6:** Annual spatiotemporal scan results for *S. enterica* serovar Typhimurium infections in Liaoning Province, 2014–2024.

Cluster type	Cluster city	Occurrence time (year)	Cluster radius (km)	RR value	LLR value	*p*-value
Class I	Chaoyang city	2014–2024	0	11.50	17.35	<0.001

Both predominant serovars showed independent high-risk clusters in Chaoyang City. A radius of 0.00 km indicates that clustering was confined to a single prefecture-level city, implying a limited spatial extent but noteworthy epidemiological relevance. This signal may be related to local food safety management, environmental sanitation, dietary habits, and/or concentrated testing practices in healthcare settings. However, the cluster could also be influenced by sentinel site configuration, testing intensity, and differences in data completeness, and therefore should not be directly equated with a true population-level high-incidence area.

Overall, the spatiotemporal scan suggests small-area, high-risk localized clustering for different Salmonella serovars in Liaoning Province and highlights the need for geographically tailored public health interventions, such as strengthening food safety supervision, environmental hygiene management, and case investigation in Chaoyang City.

Given the limited number of positive cases and the use of city-level aggregated data, these spatiotemporal clusters more likely reflect relatively higher detection levels in Chaoyang City under the sentinel surveillance framework and population distribution assumptions. Chaoyang City can be considered a potential priority area for enhanced monitoring, but further verification is needed using finer spatial scales and longer time series.

The RR indicates the relative risk within the cluster compared with outside the cluster under the discrete Poisson model. LLR quantifies the strength of evidence for the most likely cluster; larger LLR values indicate stronger clustering signals. In this study, both predominant serovars showed Class I clusters in Chaoyang City, with a radius of 0.00 km, suggesting clustering confined to a single city-level unit. Although spatial extent was small, statistical evidence was strong, highlighting the importance of enhanced monitoring and targeted interventions.

## Discussion

According to the WHO report on the global burden of foodborne diseases (2010), salmonellosis accounts for approximately 4 million disability-adjusted life years (DALYs) due to gastroenteritis, representing 22.2% of the burden attributable to microbial causes of foodborne gastroenteritis (10). Using sentinel hospital surveillance data from Liaoning Province (2014–2024), we comprehensively characterized temporal trends, seasonality, serovar distribution, and spatiotemporal patterns of foodborne salmonellosis.

The overall positivity rate was 0.89%, which differs from reported levels in several Chinese provinces, such as Shanxi (2.04% during 2014–2016) (11), Jiangsu (2.8% during 2014–2020) (12), and Hunan (9.75% during 2014–2019) (13). Such differences may reflect variation in sentinel site layouts, testing strategies, laboratory capacity, and healthcare-seeking behavior. The observed upward trend should therefore be interpreted cautiously, as it likely reflects a combination of epidemiological changes and surveillance-related factors rather than a simple increase in disease burden.

International surveillance data indicate that salmonellosis has remained a substantial public health burden in Europe and North America, with a rebound in case counts after the COVID-19 pandemic. In the EU/EEA, case numbers in recent years have approached pre-pandemic levels, and some countries have reported significant increases during 2019–2023. Notably, reported rates among children aged 0–4 years are substantially higher than among adults, supporting the interpretation that young children represent a stable high-risk population. These international observations are consistent with our finding that young children contribute a high proportion of sentinel-detected cases (14).

In recent years, multiple international studies have applied spatial statistics and spatiotemporal scan methods to evaluate spatial heterogeneity and clustering of salmonellosis. A study from Ontario, Canada used SaTScan to conduct spatial, temporal, and spatiotemporal scans for *S. enterica* serovar Typhimurium and serovar Heidelberg, finding that spatiotemporal clusters closely corresponded to known outbreak events; moreover, some clusters were detectable before laboratory subtyping results became available, suggesting the potential value of scan statistics for early outbreak warning (15).

In our study, the positivity rate remained low overall but showed a significant gradual increase during 2014–2024. Importantly, positivity rates in sentinel surveillance are influenced by case composition, healthcare-seeking behavior, clinical testing practices, sentinel coverage, and changes in laboratory capacity. Therefore, the observed upward trend may reflect a true increase in exposure risk related to changes in food supply chains and dietary behaviors, but it may also partly reflect improvements in surveillance sensitivity, increased testing, and enhanced laboratory capabilities. Future analyses incorporating a more stable population denominator or more detailed information on sentinel coverage would help disentangle these contributions.

Demographic analysis indicated that one-year-old children accounted for the highest proportion of positive cases, followed by children aged 2–9 years and infants <1 year. Children cared for at home comprised the largest occupational category, followed by retired individuals. These findings are consistent with international studies reporting that children under 5 years contribute a substantial proportion of cases (14), and with findings from Jiangsu Province (11.98% in infants <1 year and 23.39% in children aged 1–3 years) (12). Young children have immature intestinal barriers and immune function, increasing susceptibility to enteric pathogens. In addition, frequent hand-to-mouth behaviors, incomplete hygiene practices, and reliance on caregivers for food preparation and utensil sanitation can elevate exposure risk. In household cooking and take-out food scenarios, cross-contamination or inadequate heating may increase the likelihood of infection via the fecal–oral route.

The most commonly reported suspected food exposure categories were fruits and fruit products, cereals and cereal products, meat and meat products, and aquatic animal products. These results suggest that exposure may involve multiple commonly consumed foods. However, because exposure histories were self-reported, they are subject to recall bias and confounding by multiple foods; moreover, some cases involved mixed or unknown foods. Accordingly, these data are best interpreted as descriptive signals for risk communication rather than causal attribution of contamination sources.

Seasonality analysis further showed a typical unimodal summer–early autumn peak, with June–August as the very high-risk period and July as the peak month. This pattern is consistent with the tendency of Salmonella to proliferate rapidly under warm and humid conditions, increased pressure on cold-chain and food storage in summer, and increased consumption of raw foods and dining out. From a prevention perspective, summer and early autumn should be treated as key windows for intensified control, including stronger oversight of food procurement, cold-chain management, cooked-food retail, and household food handling, along with strengthened health education.

Serovar analysis indicated that among cases with definitive serovar identification, *S. enterica* serovar Enteritidis and *S. enterica* serovar Typhimurium predominated, suggesting that these remain priority serovars under sentinel surveillance in Liaoning Province. Exposure information in this study was self-reported and not suitable for causal inference linking specific serovars to specific foods. Nonetheless, the predominance of these two serovars is consistent with prior evidence indicating that Enteritidis and Typhimurium are among the most common serovars causing human salmonellosis globally (12). Similar patterns have been reported in China, including Hunan Province where Typhimurium predominated (68.17%) (11), and Jiangsu Province where Typhimurium (25.1%) and Enteritidis (23.49%) were the leading serovars (10). Therefore, in addition to continued attention to Enteritidis and Typhimurium, enhanced laboratory surveillance and risk assessment for serovar Dublin and other emerging serovars are warranted.

Spatial and spatiotemporal analyses showed no significant global spatial clustering at the city level, indicating the absence of a clear province-wide “high-risk belt” or “low-risk belt.” However, local spatial analysis suggested that while multiple cities showed clustering tendencies before correction, only Chaoyang City remained a significant hotspot after Benjamini–Hochberg FDR correction. Together with the Class I spatiotemporal clusters centered on Chaoyang City identified by SaTScan, Chaoyang City can be regarded as a localized area with consistently higher detection levels under the sentinel surveillance framework. This finding should be interpreted cautiously, as it may be shaped by factors such as sentinel site placement, testing intensity, data completeness, healthcare-seeking behavior, and local food safety management, rather than reflecting a true population-level high-incidence center. Therefore, spatial clusters and temporal trends identified in this study should be interpreted as patterns within the sentinel surveillance framework rather than direct evidence of true population-level risk.

### Limitations

This study has several limitations. First, the data were derived from sentinel hospital surveillance and include only patients who sought care and underwent testing, thus not covering all Salmonella infections in the population. Selection bias and underreporting are inevitable, and the number and distribution of sentinel hospitals may change over time. Consequently, positivity rate among tested cases cannot be directly interpreted as population-level incidence.

Second, positivity rate is influenced by clinical testing indications, laboratory capacity and healthcare-seeking behavior. We did not have access to stable population denominators, especially catchment populations of individual sentinel hospitals, and therefore could not estimate standardized incidence rates. This limits the comparability of our findings with those from other regions and countries.

Third, spatial and spatiotemporal analyses were based on aggregated city-level data and a relatively small number of positive cases. Although we applied Benjamini–Hochberg FDR correction to LISA *p*-values to reduce false positives, aggregation bias and model assumptions may still affect the results. All spatial and spatiotemporal clusters identified in this study should be regarded as exploratory signals rather than definitive evidence.

Fourth, incomplete serotyping and missing exposure information for some cases may have biased our estimates of serovar distribution and suspected food sources.

Fifth, due to national cartographic regulations, we were unable to include official map figures with administrative boundaries in this manuscript, which may limit the intuitive visualization of spatial and spatiotemporal patterns.

## Conclusion

Sentinel surveillance in Liaoning Province from 2014 to 2024 indicates that the detection level of foodborne salmonellosis remained relatively low overall but increased significantly and gradually over time. A unimodal seasonal pattern was observed, with a summer–early autumn peak. Young children, particularly children cared for at home, constituted the main susceptible population. *S. enterica* serovar Enteritidis and *S. enterica* serovar Typhimurium were the predominant serovars, and serovar Dublin and several other emerging serovars were also detected in recent years. Self-reported suspected food exposure categories suggested that exposure may involve multiple commonly consumed foods; changes in exposure profiles across years may provide clues for risk communication and source-tracing investigations, but further verification requires more complete exposure data and microbiological source-tracing evidence.

No significant global spatial clustering was detected at the city level; however, Chaoyang City consistently showed a relatively high burden in both FDR-adjusted LISA and SaTScan analyses, suggesting that it should be prioritized for strengthened regional surveillance and targeted interventions.

In practice, prevention and control strategies should balance the strengths and limitations of sentinel surveillance by expanding surveillance coverage, improving serotyping capacity, and enhancing data quality. In particular, intensified health education, food processing supervision, and cold-chain management are recommended during high-risk seasons and among young children. Incorporating multi-source data to develop higher-resolution spatiotemporal risk assessments may further reduce the public health burden of foodborne salmonellosis in the region.

Targeted prevention and control measures may be implemented along a “season-population-area” framework:

1. Season: May–September, especially June–August, should be treated as the key control window. Cold-chain temperature control and prevention of cross-contamination in food service settings should be strengthened, accompanied by more frequent inspection sampling and risk communication for high-risk foods.
2. Population: Young children and children cared for at home should be prioritized. Caregivers should receive practical household food safety education, including separation of raw and cooked foods, thorough heating, cleaning and disinfection of utensils and feeding bottles, and hand hygiene; hygiene management in childcare institutions should also be strengthened.
3. Area: For localized hotspot areas such as Chaoyang City, periodic audits of sentinel surveillance quality (testing volume, serotyping rate, data completeness) are recommended, along with enhanced laboratory serotyping and targeted epidemiological investigations to track recurrent predominant serovars and guide focused interventions.

### Author’s note

The “positivity rate” in this study reflects detection within the sentinel surveillance system and does not represent the population incidence in Liaoning Province.

## Data Availability

The datasets presented in this study can be found in online repositories. The names of the repository/repositories and accession number(s) can be found in the article/supplementary material.
