# Correction: Epidemiology, serovar distribution and spatiotemporal patterns of Salmonella foodborne infections in Liaoning Province, China, 2014–2024: an analysis of sentinel surveillance data

**DOI:** 10.3389/fpubh.2026.1825074

**Published:** 2026-03-24

**Authors:** Xinling Yu, Xiangyun Liu, Xiaoxiao Du, Yiming Pei, Kailin Wang, Hao Zhang, Wenli Diao

**Affiliations:** 1 Institute of Preventive Medicine, China Medical University, Shenyang, Liaoning, China; 2 China Medical University, Shenyang, Liaoning, China; 3 Liaoning Provincial Center for Disease Control and Prevention, Shenyang, Liaoning, China

**Keywords:** epidemiology, foodborne disease, Salmonella, spatial autocorrelation, spatiotemporal analysis

Affiliation “Liaoning Provincial Center for Disease Control and Prevention, Shenyang, Liaoning, China” was omitted for author Wenli Diao.

The funder “The key research and development project of Liaoning Province in 2024 (people's livelihood science and technology) - Establishment of prediction and early warning analysis model of foodborne diseases in Liaoning Province and research on key food safety technologies, such as population disease burden, 2024JH2/102500073 to Wenli Diao” was erroneously omitted.

The original article has been updated.

